# Effect of orange fruit peel extract concentration on the synthesis of zinc oxide nanoparticles

**DOI:** 10.1002/ansa.202400023

**Published:** 2024-08-28

**Authors:** Emebet Wondmnew, Getachew Tizazu

**Affiliations:** ^1^ Department of Physics Bahir Dar University Bahir Dar Ethiopia; ^2^ Department of Physics Debere Tabor University Debre Tabor Ethiopia

## Abstract

In this investigation, the impact of reducing agent concentration on the synthesis of zinc oxide nanoparticles (ZnO NPs) was examined. During the synthesis, an assessment of ionic conductivity was carried out, revealing a significant increase in conductivity prior to the introduction of the reducing agent, followed by a sharp decrease upon its addition. Characterization of the ZnO NPs involved ultraviolet‐visible spectroscopy, scanning electron microscopy, Fourier‐transform infrared spectroscopy, and, X‐ray diffraction analysis. The outcomes suggest that the characteristics of the ZnO NPs are influenced by the concentration of the reducing agent during the synthesis process. Notably, the ZnO NPs synthesized with a higher concentration of reducing agent exhibited a narrower optical band gap and increased surface energy. Furthermore, employing a concentration of 0.5 v/v resulted in the rapid production of NPs with relatively uniform sizes. Conversely, concentrations below 0.5 v/v lead to slow formation, while concentrations exceeding 0.5 v/v yielded non‐uniform NPs.

**Abbreviations**
EDX: energy dispersive X‐ray spectroscopy
FTIR: Fourier‐transform infrared
NaOH: sodium hydroxide
SEM: scanning electron microscopy
TGA: thermogravimetric analysis
UV‐Vis: ultraviolet‐visible
XRD: X‐ray diffractometer
Zn(NO_3_)_2_·2H_2_O: zinc nitrate dihydrate
ZnO NPs: zinc oxide nanoparticles

## 1 INTRODUCTION

Metal oxide nanoparticles (NPs) can be synthesized using environmentally friendly reducing agents such as plant extracts.^1^ These methods are nowadays more preferred than chemical and physical methods because they are non‐toxic and less costly.^2^ There are studies on the effect of the reducing agent concentration on the size and shape of metal NPs. Rajalakshmi et al. studied the effect of the concentration of Impatiens balsamina L. plant flower extract (utilized as a reducing agent) on the synthesis of silver NPs and observed that high concentrations of extract led to the production of smaller NPs.^3^ Similarly, Kim et al. explored variations in reducing agent concentration during the synthesis of Au NPs. Their findings indicated that an increase in reducing agent concentration resulted in a reduction in the size of AuNPs.^4^ Despite the prevalent preference for green synthesis over chemical methods, there is a notable absence of comparative research on the effects of reducing agent concentration on the characteristics of zinc oxide NPs (ZnO NPs) in the existing literature. Therefore, in this paper, we studied the effect of reducing agent concentration on the size of ZnO NPs, which are one of the most widely used metal oxide NPs for device fabrication and preparation of beauty products.^5^ In the investigation, scanning electron microscopy (SEM) analysis was used to confirm shape and size, and energy dispersive X‐ray spectroscopy (EDX) was utilized to check the purity and composition of the NPs. Fourier‐transform infrared (FTIR) spectroscopy was employed to examine the stretching and bonding, X‐ray diffraction (XRD) was used to investigate the structure and average size, and ultraviolet‐visible (UV‐Vis) was utilized to study the optical properties of the ZnO NPs.

## 2 MATERIALS AND METHODS

### 2.1 Chemicals and materials

Zinc nitrate dihydrate [Zn(NO_3_)_2_·2H_2_O, 99.5%], sodium hydroxide (NaOH, 98%), ferric chloride (FeCl_3_, 10%), sulfuric acid (H_2_SO_4_, 98%), ethanol (98%), Wagner's reagent, and hydrochloric acid (HCl, 35.4%) were obtained from Abron. The orange fruit was purchased from the local market in Bahir Dar City, Ethiopia. All glassware was thoroughly cleaned with deionized water and ethanol and then dried in an oven before use.

### 2.2 Preparation of orange fruit peel extract

**FIGURE 1:**
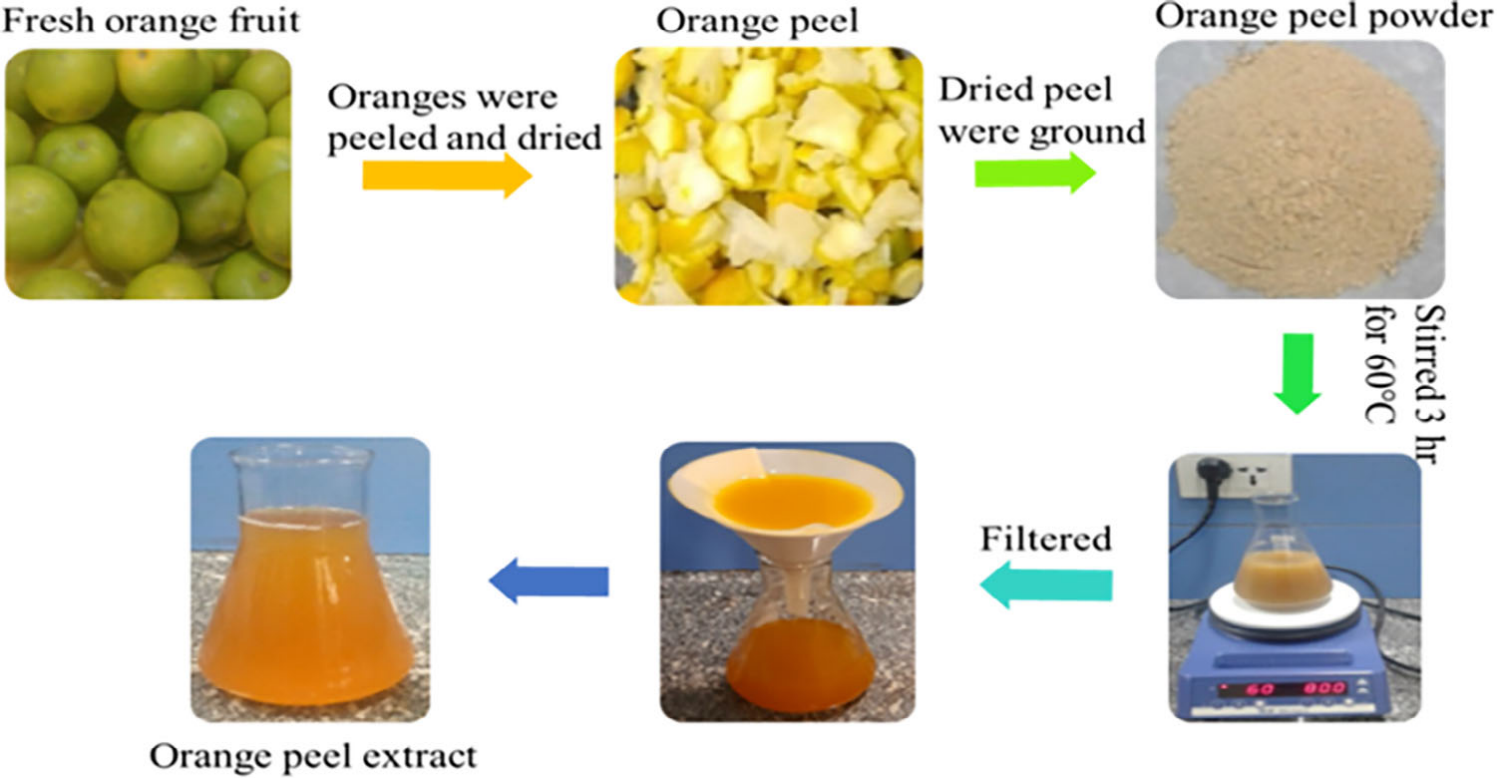
A photograph that shows the steps involved in the preparation of orange peel extract.

To obtain the extracts, the orange fruit was repeatedly washed and then carefully peeled as thinly as possible. The peels were dried in an oven at 60°C for 24 h until fully dehydrated and then ground into a moderately fine powder. A 50 g portion of the orange peel powder was added to 250 mL of deionized water and stirred using a magnetic stirrer while being heated to 60°C for 3 h on a hot plate (IKA RH digital). After cooling to room temperature for approximately 15 min, the solution was filtered twice using Whatman filter paper. The general procedure for preparing the orange peel extract is illustrated in Figure 1.

### 2.3 Phytochemical test of orange fruit peel extract

Phytochemical tests were conducted to detect the presence of flavonoids, polyphenols, alkaloids, tannins, and glycosides in the extract. The complete procedure used for the phytochemical testing of orange peel extract is detailed in Figure S1.

### 2.4 Synthesis of ZnO NPs

Zn(NO_3_)_2_·2H_2_O (10 g) was dissolved in 500 mL of deionized water to create a 0.1 M solution. The solution was continuously stirred for 20 min using a magnetic stirrer to ensure the complete dissolution of the zinc nitrate. The influence of orange peel extract concentration on the characteristics of the ZnO NPs was studied by varying the ratio of orange peel extract to zinc nitrate dihydrate solution. Accordingly, samples A, B, C and D were prepared by adding 25 mL (0.25 v/v), 35 mL (0.35 v/v), 50 mL (0.5 v/v), and 100 mL (1 v/v) of orange peel extract to 100 mL of zinc nitrate dihydrate solution, respectively. The pH of the mixture was increased to 12 by gradually adding a 2.0 M NaOH solution while stirring and heating the mixtures at 60°C for 2 h using a magnetic stirrer. Stirring continued for an additional 30 min until a solid product of a pale cream colour was achieved. To eliminate any residual impurities or unreacted components, the precipitate was washed three times with ethanol and deionized water. Subsequently, the precipitate was dried in an oven (model: Ambala Cantt‐133001 HR) at 60°C for a duration of 36 h to ensure complete drying. The resulting product was then subjected to calcination using a Muffle furnace (model: ME2.5‐12GJ) at 400°C for 2 h.

**FIGURE 2:**
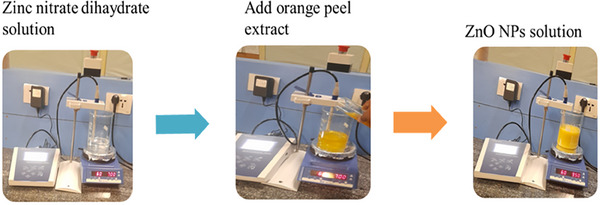
A photograph of the conductivity measurement setup during the synthesis of zinc oxide nanoparticles (ZnO NPs).

To confirm the formation of NPs, visual observation was first conducted. Additionally, conductivity measurements were performed using a conductivity meter (model; 4510, Jenway). In this process, 100 mL of zinc nitrate dihydrate solution was first placed in a beaker and its conductivity was recorded, see Figure 2. Then, while the conductivity meter remained in the solution, 50 mL of orange peel extract was added. Conductivity was continuously monitored while the mixture was stirred at 60°C with a magnetic stirrer.

The optical properties of the ZnO NPs, synthesized with varying ratios of orange peel extract and zinc nitrate solution, were measured using a UV‐Vis spectrometer (DR6000 model). The band gap energy of the ZnO NPs was determined from the UV‐Vis spectra using the Tauc plot methodology.^6^ Additionally, the NP size was calculated from the band gap using the effective mass mode (Equation (1)).^7^

(1) $$\begin{array}{cc} r & =\frac{2A}{-B+\sqrt{B^{2}+4AC}}\;\mathrm{where}\;A\;=\frac{h^{2}}{8m_{0}e^{2}}\;\left(\frac{1}{m_{e}^{*}}+\;\frac{1}{m_{h}^{*}}\right),\\ B & =\;-\frac{1.8e^{2}}{4\pi \varepsilon \varepsilon_{0}},C\;=E_{g}^{bulk}\;-E_{g}^{*}-\frac{0.124e^{3}m_{0}}{h^{2}{\left(2\varepsilon \varepsilon_{0}\right)}^{2}}{\left(\frac{1}{m_{e}^{*}}+\;\frac{1}{m_{h}^{*}}\right)}^{-1} \end{array}$$

where $E_{g}^{Bulk}$ is the bulk band gap of ZnO (3.2 eV),^8^ $h=6.625\times 10^{-34}J\cdot s$, *r* is particle radius (m), $\;m_{o}$ is the free electron mass $\left(9.11\times 10^{-31}\mathrm{kg}\right)$, $m_{e}^{*}$ is effective mass of an electron $\left(0.24m_{o}\right)$, $m_{h}^{*}$ is effective mass of the hole $\left(0.45m_{o}\right)$, *e* is the charge on an electron $\left(1.602\times 10^{-19}C\right)$, $\varepsilon_{0}$ is the permittivity of free space (8.85 × 10^−12^ C^2^ m^−2^), and ɛ is relative permittivity (3.7).^3^

Additionally, the concentration of the NPs was determined from the same UV‐Vis spectra using the Beer‐Lambert law, A=αcl, where A is absorbance, α is the molar extinction coefficient (in M^−1^ cm^−1^), l is the path length of the sample (1 cm), and c is the concentration of the NPs (M).^9^ The presence of capping agents on the surface of the ZnO NPs was analyzed using FTIR spectra (Perkin Elmer Spectrum two spectrometer). In order to take FTIR spectra, the ZnO NPs were made into pallets using KBr and a pallet‐making machine. The crystallinity of the NPs was investigated by acquiring XRD patterns with an X‐ray diffractometer (XRD‐6000, Shimadzu, Japan) using Cu Kα radiation (λ = 0.15406175 nm). Based on the XRD data, the crystalline size was determined using the Debye‐Scherer equation, $D=\frac{K\lambda }{\beta cos\;\theta }$ where, D is the average size of the crystalline, λ is the wavelength of the X‐ray (0.15406175 nm), k is the shape factor or Scherer's constant (0.94), θ is Bragg's diffraction angle, and β is the full width at half maximum of the XRD peak.^10^ Moreover, the morphology and particle size were studied using a scanning electron microscope (JEOL JSM‐6701F) and the elemental composition was determined using an EDX (ZEISS SIGMA). The thermal stability was analyzed by thermogravimetric analysis (TGA, Bjhenven, HCT‐1). The surface energy of the ZnO NPs, $\gamma_{nano}=\;\gamma_{0}\;{\left(1-\;\frac{r_{o}}{r}\right)}^{2},$ was calculated based on the size (r), bond energy, and lattice constants of the unit cell.^11^

## 3 RESULTS AND DISCUSSIONS

**FIGURE 3:**
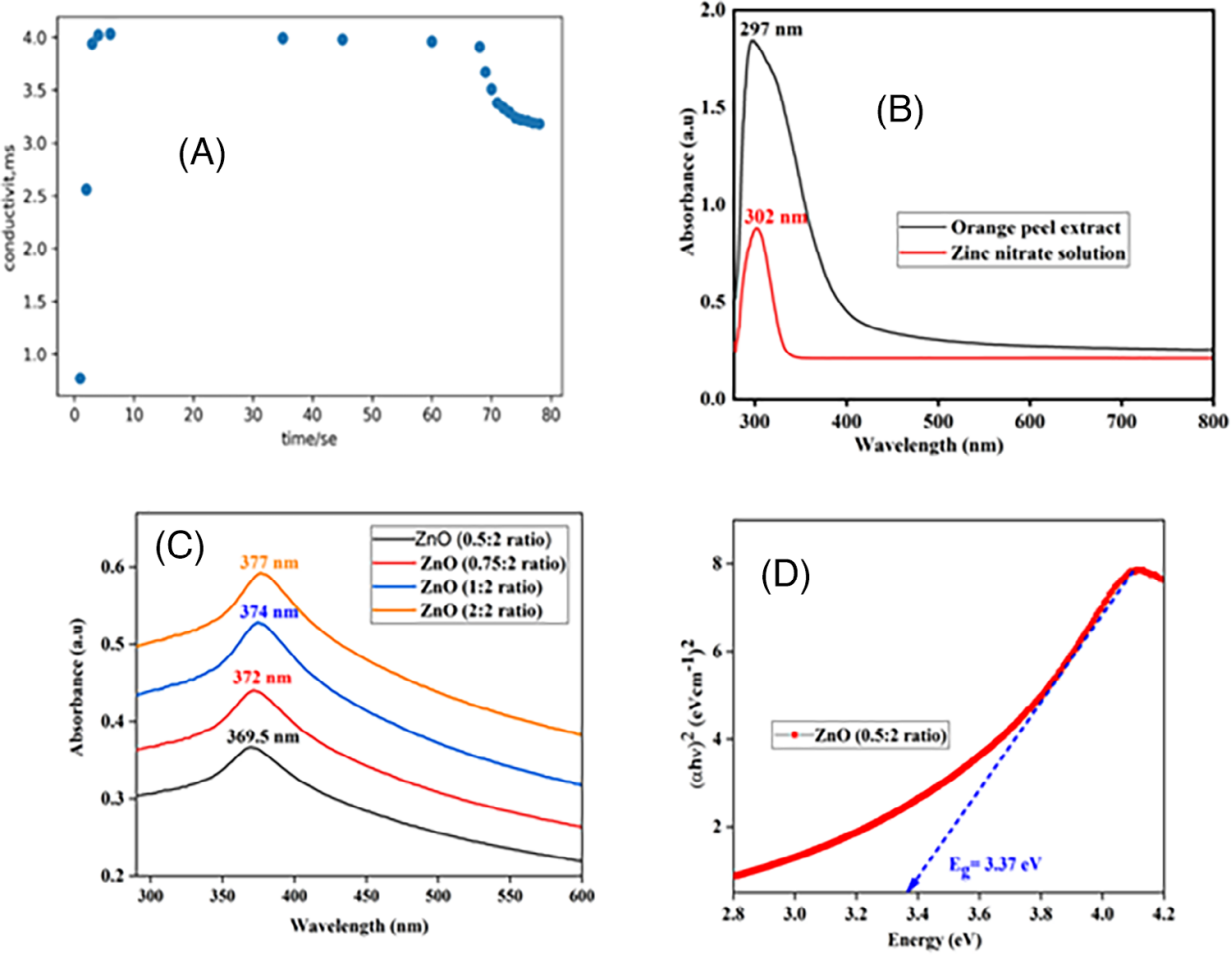
(A) Plot of conductivity versus time of reaction. (B) Ultraviolet (UV)–visible absorption spectra of orange peel extract and zinc nitrate solution. (C) UV‐Vis spectra of the zinc oxide nanoparticles (ZnO NPs) synthesized using different concentrations of orange peel extract as a reducing agent and (D) the Tauc plot of the ZnO NPs synthesized using a concentration of 0.25 v/v orange peel extract.

Figure 3A shows the variation in the conductivity of the ZnO NPs forming mixtures over time. Initially, there was a sharp increase in conductivity that was attributed to the dissolution of the precursor (zinc nitrate), leading to an increase in the number of ions in the solution. Subsequently, a plateau in conductivity was observed between 5 and 60 s, indicating complete dissolution of all precursors with no further ion addition. In the final stage, a decrease in conductivity was noted because of the introduction of a reducing agent, which facilitated the reduction of ions to neutral NP‐forming atoms.

Figure 3B shows the UV–vis absorbance spectra of both the orange peel extract and the zinc nitrate solution, with the orange peel extract displaying a peak at 297 nm and the zinc nitrate solution at 302 nm. In Figure 3C, the UV–vis absorbance spectra of ZnO NPs, recorded between 290 and 600 nm, are presented. The peak positions in these spectra vary according to the amount of extract used during the synthesis of the ZnO NPs. These spectra were employed to determine the band gap using the Tauc equation^12^ (Figure S3). The calculated band gap values were 3.37, 3.34, 3.33, and 3.30 eV for extract concentrations of 0.25, 0.35, 0.5, and 1, respectively, as illustrated in Figure S4 and Table S2.

**FIGURE 4:**
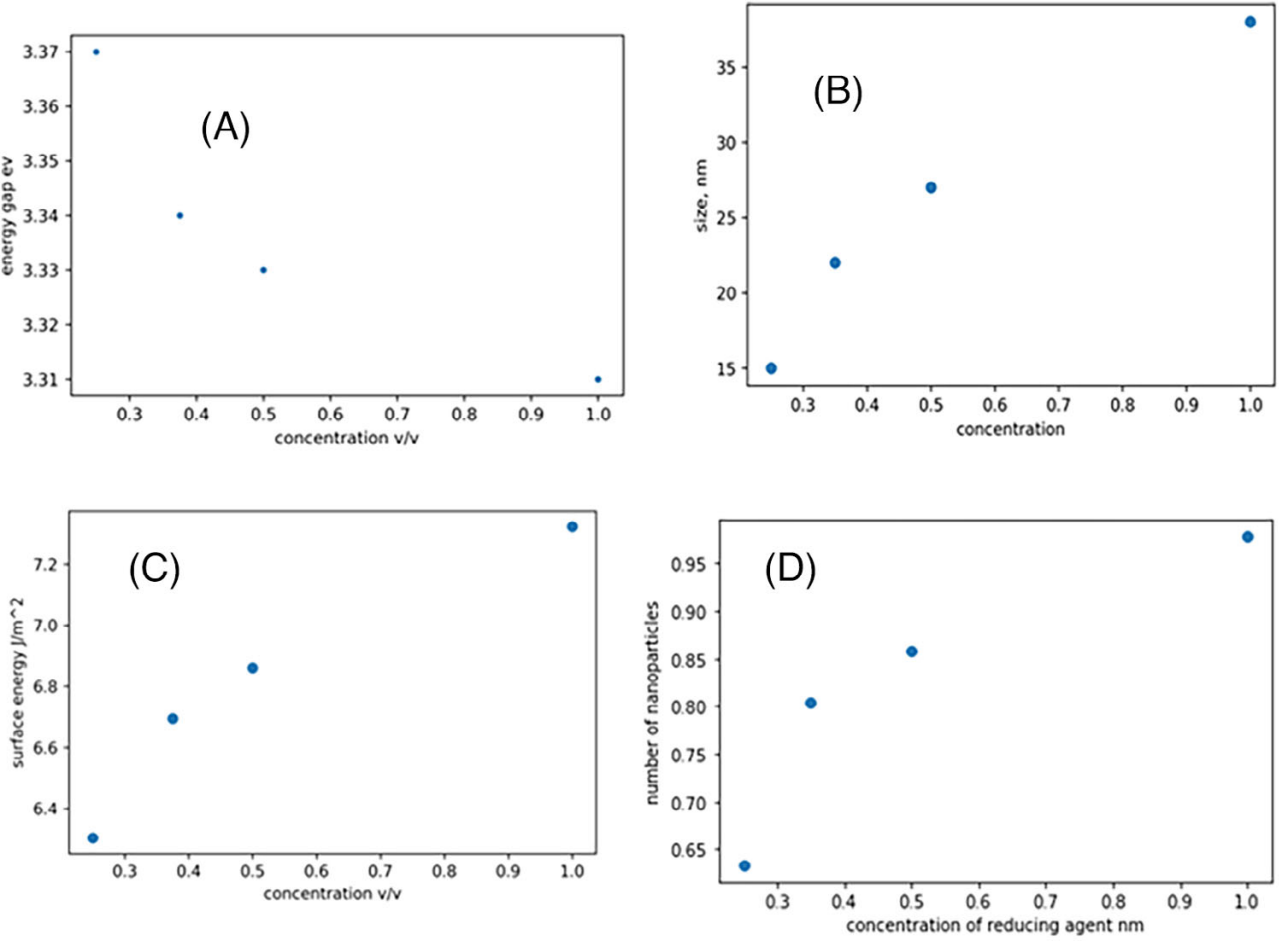
(A–D) plot of optical bandgap, size, surface energy, and number of zinc oxide nanoparticles(ZnO NPs) versus concentration of the reducing agent.

Figure 4A–D are plots of the band gap, size, surface energy, and concentration of the ZnO NPs against the concentration of the extract utilized during the synthesis process. The decrease in the band gap as orange peel extract increases can be due to the increase in the size of the NPs.^13^ As the concentration of orange peel extract increases, the availability of electron donors rises due to the higher concentration of the reducing agent. These electron donors assist in reducing precursor ions to their corresponding neutral atoms, which accelerates the surface reaction rate and results in the formation of larger NPs. In addition to the band gap, the peak wavelength was also determined from the UV‐Vis spectra and plotted, as shown in Figure S4. The peak wavelength shifts toward longer wavelengths with increasing reducing agent concentration, further indicating the formation of larger NPs as the amount of reducing agent increases.^14^ In Figure 4C, the observed increase in surface energy with increasing reducing agent concentration can be attributed to the subsequent increase in size leading to a larger surface area and ultimately higher surface energy.

The peaks on UV‐Vis spectra can also be utilized to calculate NP concentrations via the Beer‐Lambert law (Equation s6). To accurately calculate the concentration as per Equation s6, it is essential to have knowledge of the molar extinction coefficient specific to the NPs under consideration. The extinction coefficient has been determined for ZnO NPs in correlation with their particle size. The relevant data was extracted^15^ and it was replotted using matplotlib, (Figure S2) and a mathematical correlation between the extinction coefficient and particle size was established (see Equation s7). Using this relation, the number of ZnO NPs was determined and the calculated number of NPs was plotted against orange peel extract as shown in Figure 4D. The plot shows a rapid increase in the number of ZnO NPs as the concentration of the reducing agent increases from 0 to 0.35 v/v; however, after 0.35 v/v, the rate of change decreases. This phenomenon can be attributed to the critical role that zinc ions and the reducing agent play in the production of ZnO NPs. Manipulation of only one factor, such as the concentration of the reducing agent in this scenario, can significantly impact the rate of NP formation, provided that a sufficient quantity of zinc ions is available for reduction. Conversely, if the concentration of the reducing agent increases without a corresponding increase in zinc ions, there will be no significant influence on the formation rate.

**FIGURE 5:**
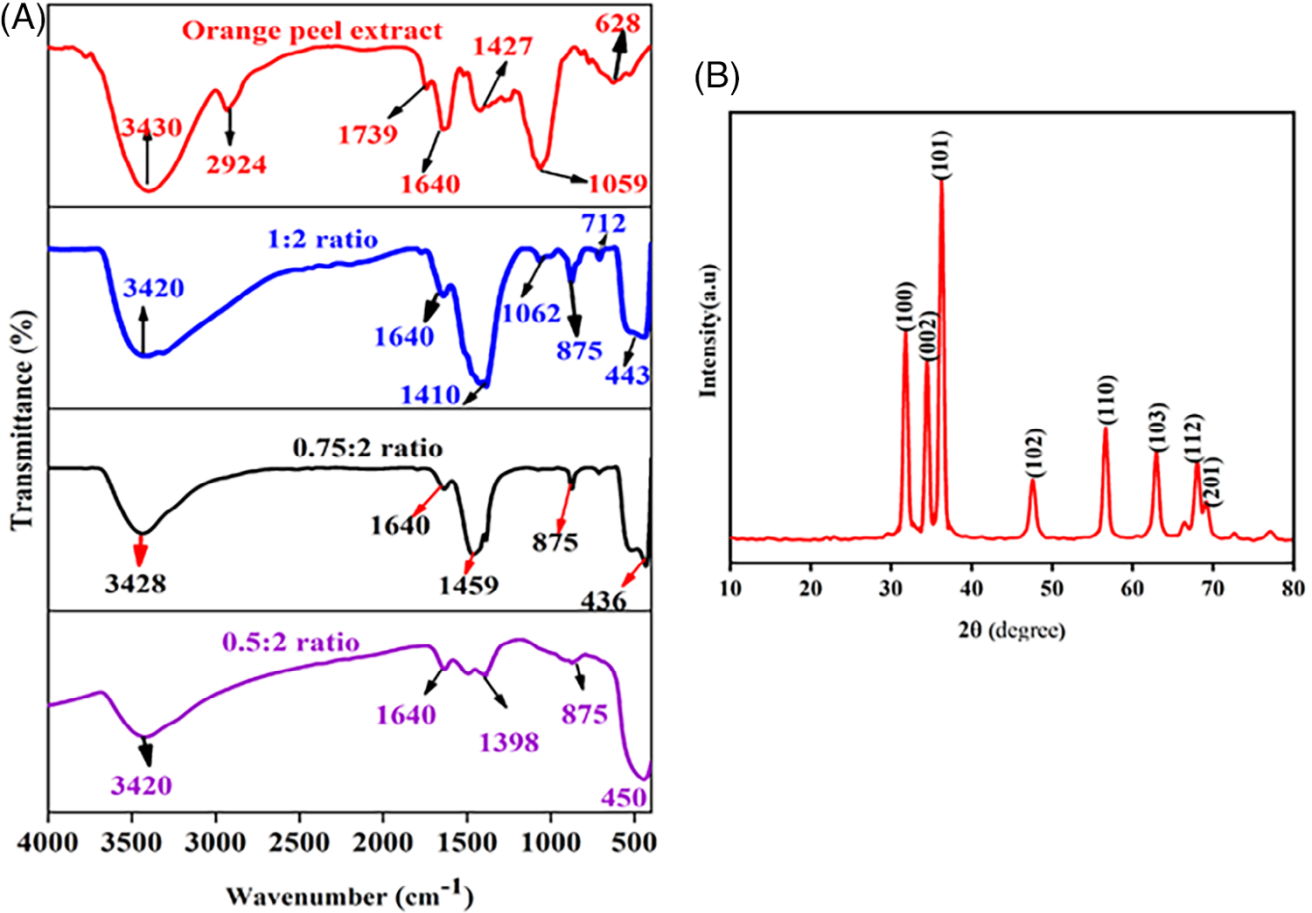
(A) Fourier‐transform infrared (FTIR) spectra of aqueous orange peel extract and zinc oxide nanoparticles (ZnO NPs) and (B) X‐ray diffraction (XRD) pattern of the ZnO NPs.

Figure 5A shows the FTIR spectra of ZnO NPs synthesized with different proportions of orange peel extracts. The FTIR spectra of alcohols, phenols, or water molecules exhibit a significant peak around 3414–3442 cm^−1^ attributed to O‐H stretching. Peaks within the range of 1400–1649 cm^−1^ are linked to C = O stretching, while the band at 1398 cm^−1^ is associated with the bending vibration of COH. Furthermore, Zn‐OH^16^ stretching vibrations are denoted by small, intense bands at 875 and 712 cm^−1^. The detailed peak positions and their corresponding vibrational modes are summarized in Table S3, Figure 5B presents the XRD pattern of ZnO NPs synthesized using 0.5 v/v of orange peel extract. The diffraction peaks confirm the formation of ZnO NPs with zincate phases, exhibiting a hexagonal wurtzite crystal structure. The lattice constants were determined to be a = b = 3.248532 and c = 5.203366. The estimated average crystalline size (D) of the synthesized ZnO NPs was approximately 19.2 nm. The crystalised size of the individual peaks is presented in Table S4.

**FIGURE 6:**
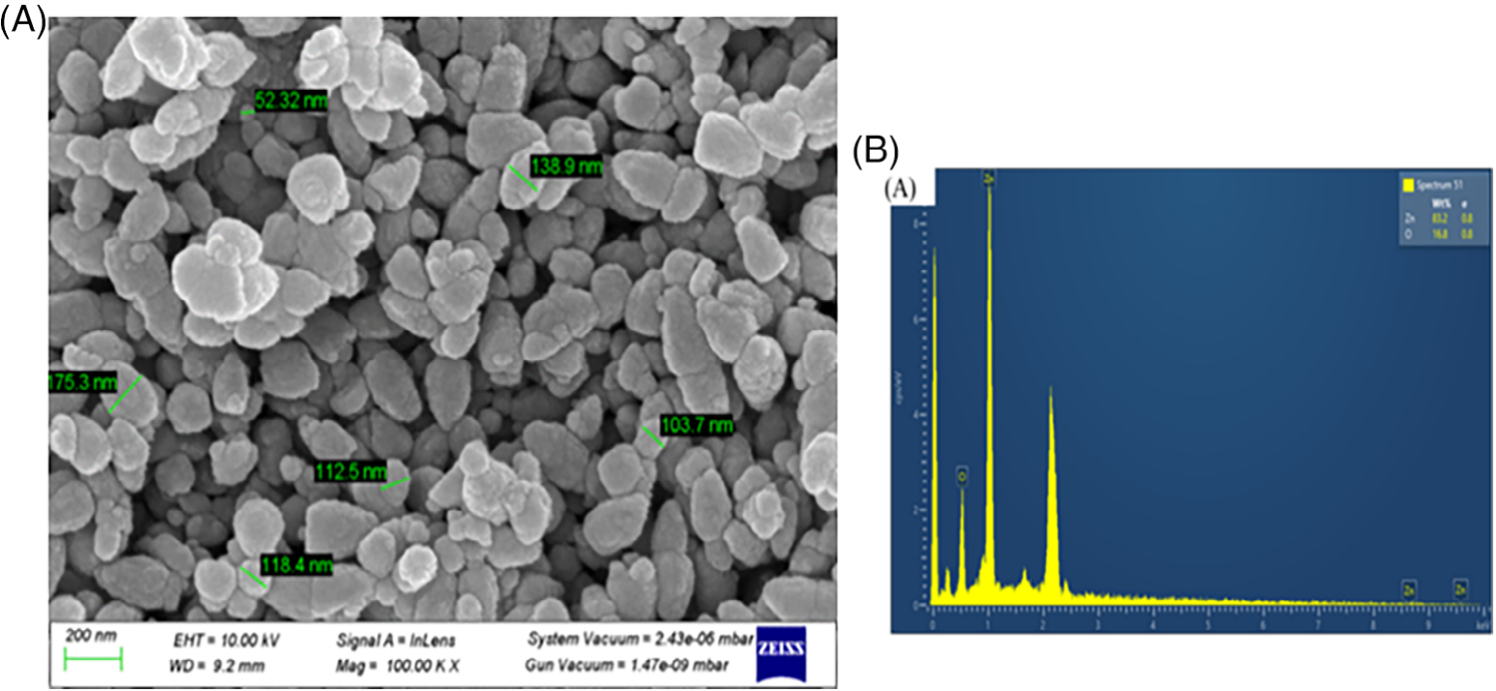
(A) Scanning electron microscopy (SEM) image of the zinc oxide nanoparticles (ZnO NPs) synthesized using 0.5 v/v orange peel extract and (B) elemental analysis of the same sample with energy dispersive X‐ray spectroscopy (EDX).

The morphology of the ZnO NPs was revealed by SEM analysis, as depicted in Figure 6. The particle sizes observed in the SEM images ranged from 75 to 180 nm with an average particle size of 100 nm, demonstrating consistency with prior research outcomes.^17^ Discrepancies in the average particle size as presented in Figure 4B, derived from the Uv vis spectroscopy measurement (70 nm) and SEM (100 nm), might be attributed to the prolonged storage period (90 days for SEM and merely one day for UV‐Vis measurement) prior to the SEM analysis, potentially resulting in particle aggregation and coalescence.

Elemental analysis was performed using EDX (Figure S5), and the existence of zinc was confirmed by the presence of a few peaks between 1 and 10 Kev, including a significant peak at 1 keV (Figure 6B). The zinc and oxygen elements are present with a weight percentage of 83.22% and 16.78% (see Table S5), respectively, which is close to the bulk weight percentage of ZnO (80 for Zn and 20 for O). In addition, the analysis showed atomic percentages of 54.82% for zinc and 45.18% for oxygen, with an atomic percentage composition similar to the results reported in related studies.^18^

**FIGURE 7:**
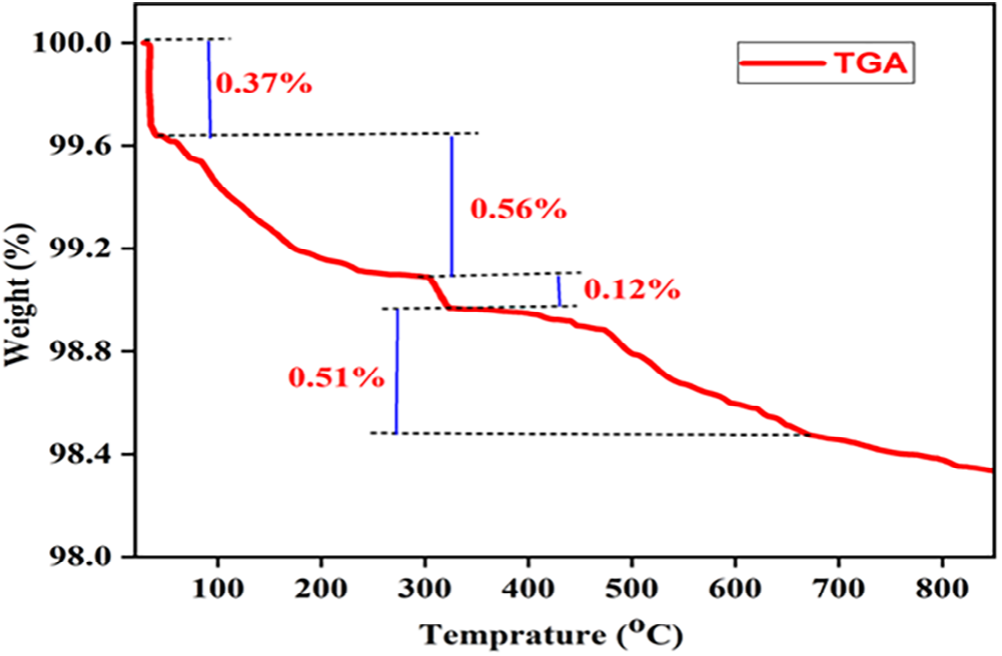
Weight loss versus temperature plot of zinc oxide nanoparticles.

The thermal stability of ZnO NPs was investigated using TGA (BJHENVEN, HCT‐1) with a scan rate of 15°C/min in the range of 20‐800°C under ambient conditions. The weight loss plot against temperature for the ZnO NPs is shown in Figure 7. The graph illustrates four distinct weight loss phases within the defined temperature range. The initial decomposition, which occurs between 30°C and 60°C with a weight loss of 0.37%, is related to the elimination of surface impurities. The subsequent phase, observed from 60°C to 305°C with a weight loss of 0.56%, is attributed to the evaporation of water molecules and the dehydration of hydroxyls. The third phase, which spanned 305°C to 326°C, resulted in a weight loss of 1.12%, possibly due to the breakdown of the organic compounds that protect the synthesized ZnO NPs. The final phase of weight loss is associated with the decomposition of any residual organic components or the conversion of specific inorganic species present in ZnO NPs at temperatures ranging from 326°C to 670°C, leading to a weight loss of 0.5%. After 670°C, no substantial weight loss was observed, which confirms the thermal stability of the ZnO NPs, consistent with findings reported in other literature.^19^

## 4 CONCLUSIONS

In the present study, the observed decrease in conductivity during the synthesis process provides preliminary evidence for the reduction of zinc ions and the potential formation of ZnO NPs. UV‐Vis spectral analysis confirmed that the maximum absorption was in the 365–380 nm range, which is specific for ZnO NPs. Furthermore, the optical band‐gap energies were determined from UV‐Vis spectroscopic data using the Tauc plot. An increase in the concentration of the reducing agent resulted in a decrease in the ZnO NPs band gap and a shift in the absorption maxima toward higher wavelengths. FTIR analysis showed characteristic peaks at 436, 443, and 450 cm^−1^, confirming the formation of ZnO NPs. XRD analysis confirmed the hexagonal wurtzite structure of the ZnO NPs, with well‐matched diffraction peaks. The SEM analysis confirmed the size and morphology of the NPs. The elemental composition, elemental mapping, and purity were determined by EDX studies.

## AUTHOR CONTRIBUTIONS

Emebet Wondmnew conducted the experimental work and prepared the draft paper while Getachew Tizazu conceived the idea and wrote the final paper.

## CONFLICT OF INTEREST STATEMENT

The authors declare no conflicts of interest.

## FUNDING INFORMATION

The authors acknowledge the International Science Program (ISP), Sweden, for financial support. Emebet Wondmnew also expresses gratitude to Debre Tabor University for providing financial support for her master's degree.

## Supporting information

Supporting information

## Data Availability

The data that support the findings of this study are available on request from the corresponding author.
