# Supplementary material for: Effect of orange fruit peel extract concentration on the synthesis of zinc oxide nanoparticles
Source: Anal Sci Adv. 2024 Aug 28;5(7-8):e2400023. doi: 10.1002/ansa.202400023 (PMC11361366; doi:10.1002/ansa.202400023)
Supplement: Supplementary file 1 — Supporting information [file ANSA-5-e2400023-s001.docx]

**Effect of Orange Fruit Peel Extract Concentration on the Synthesis of Zinc Oxide Nanoparticles**

# Emebet Wondmnew and Getachew Tizazu^*^

Department of Physics, Bahir Dar University, Bahir Dar, Ethiopia

*Email [getachewtizazu@gmail.com](mailto:getachewtizazu@gmail.com)

**Supporting information**


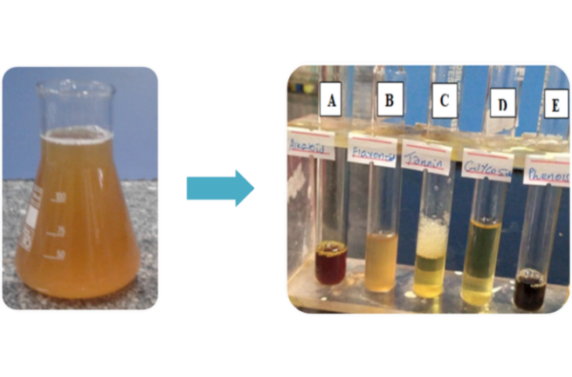
**Phytochemical tests:** Figure 3 illustrates the phytochemical tests of the orange peel extracts where A, B, C, D, and E corresponds to a screening for alkaloids, flavonoids, tannins, glycosides, and phenols, respectively. Table 1 is the results of the phytochemical examination. The test has yielded evidence that the orange peel extract contains phytochemicals capable of reducing zinc. Furthermore, these phytochemicals may encapsulate and stabilize zinc oxide nanoparticles.

Figure s1: a) Orange fruit peel extract and b) test tubes for phytochemical screening where A, B, C, D, and E corresponds to a screening for alkaloids, flavonoids, tannins, glycosides, and phenols, respectively

Table s1: Results of phytochemical screening of orange peel extracts.


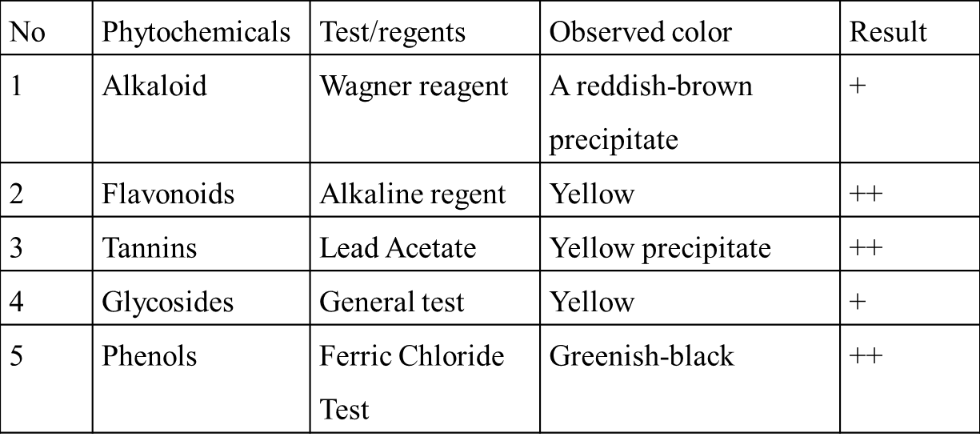


Keys: ++ = Present in excess, + = Present in excess

**Size Determination of the ZnO NPs from UV Vis Spectroscopy:** Before determining the size, the band gap of the ZnO NPs was determined through the utilization of the Tauc plot in UV Vis spectroscopy. Subsequently, the size of the ZnO NPs was derived from the band gap by employing the effective mass model (Equation s1). After solving Equation s1, the size of the ZnO NPs can be obtained using Equation s2.

$E_{g}^{*}= E_{g}^{bulk}+ \frac{h^{2}}{8{m_{0}e r}^{2}}\left( \frac{1}{m_{e}^{*}}+ \frac{1}{m_{h}^{*}} \right)-\frac{1.8e^{2}}{4\pi\varepsilon\varepsilon_{0}r} - \frac{0.124e^{3}m_{0}}{h^{2}\left( 2\varepsilon\varepsilon_{0} \right)^{2}}\left( \frac{1}{m_{e}^{*}}+ \frac{1}{m_{h}^{*}} \right)^{-1}$……..s1

$r=\frac{2A}{-B+\sqrt{B^{2}+4AC}}$ ……………………..…………………………………………. s2

Where $A=\frac{h^{2}}{8{m_{0}e}^{2}}\left( \frac{1}{m_{e}^{*}}+ \frac{1}{m_{h}^{*}} \right)$, $B=-\frac{1.8e^{2}}{4\pi\varepsilon\varepsilon_{0}}$, $C=E_{g}^{bulk}-E_{g}^{*}-\frac{0.124e^{3}m_{0}}{h^{2}\left( 2\varepsilon\varepsilon_{0} \right)^{2}}\left( \frac{1}{m_{e}^{*}}+ \frac{1}{m_{h}^{*}} \right)^{-1}$

Where, $E_{g}^{Bulk}$is the bulk band gap of ZnO (3.2eV)^1^, $h$ =$6.625\times10^{-34}\text{ J}\cdot\text{s}$, *r* is particle radius (m),$m_{o}$ is the free electron mass ($9.11\times10^{-31}\text{ kg}$), $m_{e}^{*}$ is effective mass of a conduction band electron in ZnO (0.24$m_{o}$), $m_{h}^{*}$ is effective mass of a valence band hole in ZnO (0.45$m_{o}$ ),*e* is the charge on an electron($1.602\times10^{-19}\text{C}$), $\varepsilon_{0}$ is the permittivity of free space ($\in_{0}$), $\in$ is relative permittivity of zinc oxide (3.7) ^2^.

**Refractive index :** the refractive index of materials can be determined from the energy gap using the Herve and Vandamme relation ^3^.

$n=\left( 1+\left( \frac{13.16 eV}{E_{g}+3.17 eV} \right)^{2} \right)^{1/2}$ s3

Where n is refractive index, and Eg is band gap.

**Determination of Surface Energy**: The estimation of surface energy for ZnO NPs can be readily calculated based on the bond energy, bulk surface energy, and lattice constants of the unit cell^4^. The correlation among bulk surface energy, lattice parameter, and bond energy is defined by equation s4. Furthermore, the calculation of surface energy for nanoparticles with radius r is achievable through equation s5. The numerical values of the constants include Avogadro’s number (N_A_=6.02214 x 10^23 mol^-1^), lattice parameter (a) for zinc oxide is 0.32 nm, critical radius r_0_ for zinc oxide nanoparticles is 1.6 nm, and bond energy of zinc oxide (E_av_) is 284.1 kJ/mol^5^.

$\gamma_{0}=\frac{\sqrt{3}}{N_{A}a^{2}}E_{av}$ ………………………………………….s4

$\gamma_{nano}=\gamma_{0}\left( 1- \frac{r_{o}}{r} \right)^{2}$……………………………………….s5

**Determination of concentration of ZnO NPs:**  The number of ZnO NPs was estimated from UV-vis spectroscopy using the Beer-Lambert law, see Equation s6^6^. Furthermore, the uniformity of the ZnO NPs was estimated from the FWHM of the UV-vis spectra.

$A= \alpha cl$………………………………..s6

Where A is absorbance, α is the molar extinction coefficient with unit of M^−1^ cm^−1^, l is the path length of the sample (1 cm), and c is the number of suspensions of ZnO NPs (M).

To accurately calculate the concentration as per Equation s6, it is essential to have knowledge of the molar extinction coefficient specific to the nanoparticles under consideration. The extinction coefficient has been determined for ZnO NPs in correlation with their particle size. The relevant data was extracted from ref^7^ and it was replotted using matplotlib, see Figure s2. The fitting process was carried out utilizing SymPy optimize, to establish a mathematical correlation between the extinction coefficient and particle size. The fit equation is ε=0.02564 r^3^ + 0.02051 r^2^ - 0.2084 r + 1.511 with R square value of 0.99 and the number of nanoparticles can be estimated using equation s7.


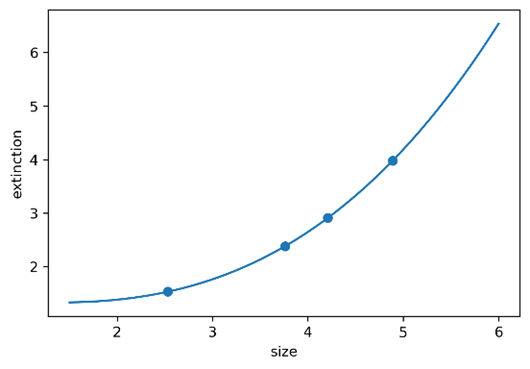
$n=\frac{A}{(0.02564 r^{3} + 0.02051r^{2} - 0.2084 r + 1.511)l}$ s7

Figure s2: Plot of the extinction coefficient versus the size of the ZnO NPs.

**
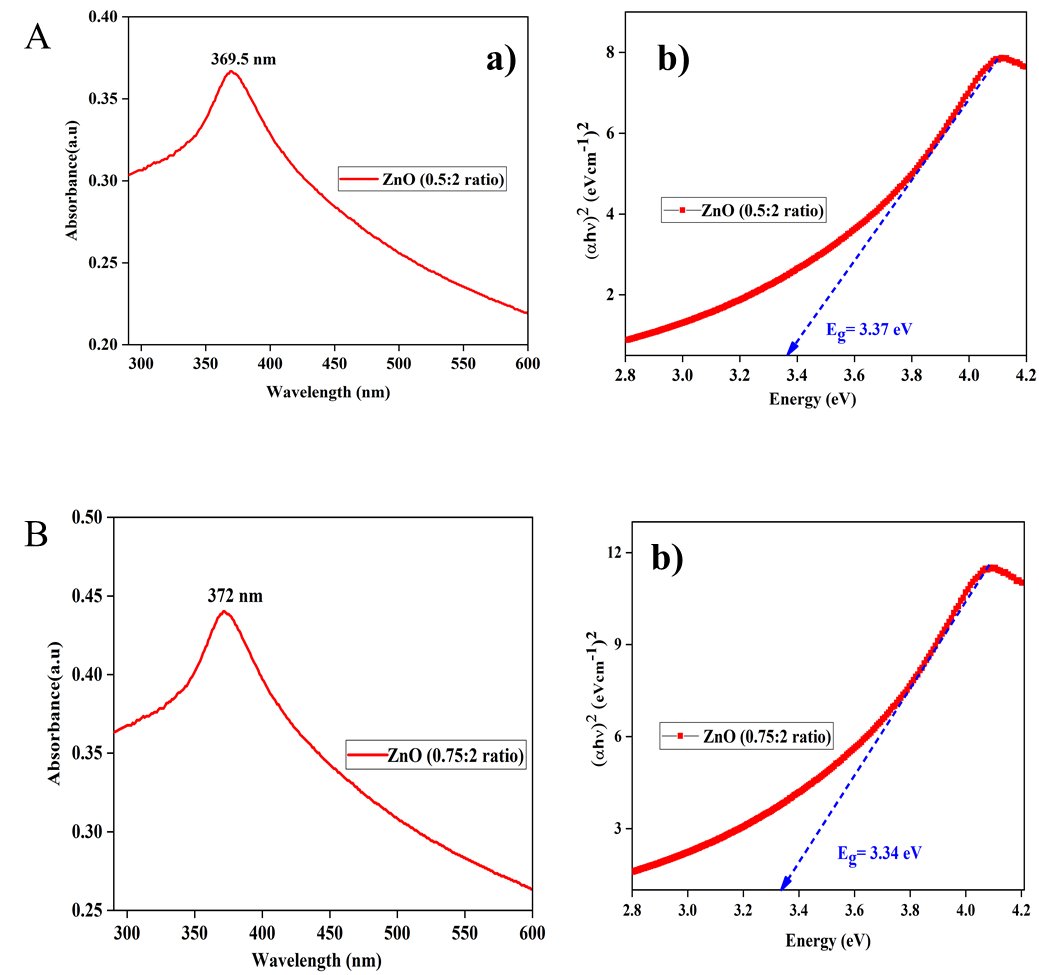
Band gap determination:** The band gap of the nanoparticles was determined using the Tauc plot. Figure s3 is the UV-Vis absorption spectrum of ZnO NPs alongside the corresponding Tauc plot. Specifically, sample A is associated with a ratio of 0.5:2, sample B with a ratio of 0.75:2, sample C with a ratio of 1:2, and sample D with a ratio of 2:2.


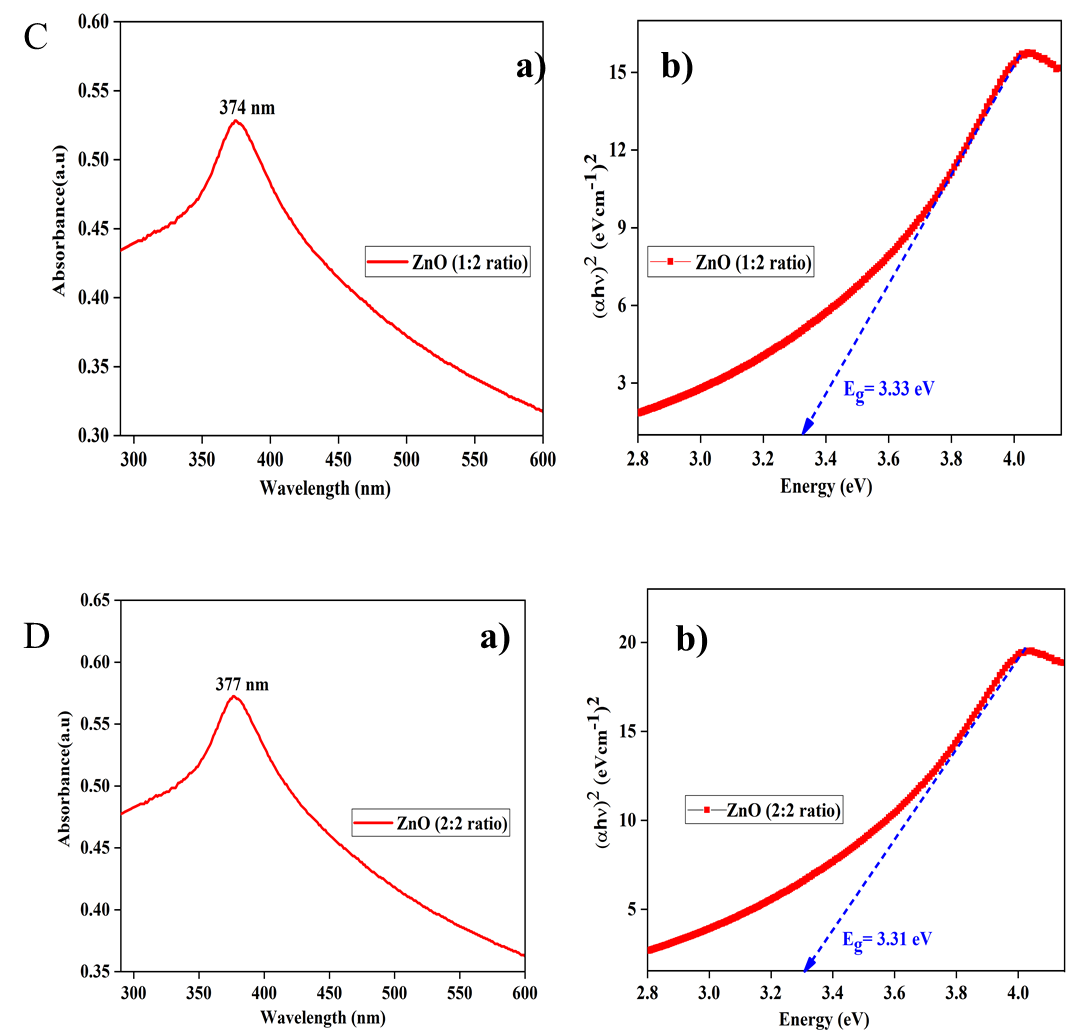


Figure s3. Uv vis absorption spectrum of ZnO NPs alongside the corresponding Tauc plot. Specifically, sample A is associated with a ratio of 0.5:2, sample B with a ratio of 0.75:2, sample C with a ratio of 1:2, and sample D with a ratio of 2:1.

Table s2: Energy band gap of the ZnO NPs for different concentration of orange peel extract.

| No | ZnO NPs with different concentration of orange peel extract | Stirring time, hrs. | | Solution T℃ | Calcination T℃ | Wavelength (nm) | Band gap (eV) |
| --- | --- | --- | --- | --- | --- | --- | --- |
|  |  | Before adding NaOH | After adding NaOH |  |  |  |  |
| 1 | 0.25 (0.5:2 ratio) | 2hr | 0.3 min | 60℃ | 400℃ | 369.5 nm | 3.37eV |
| 2 | 0.35 (0.75:2 ratio) | 2hr | 0.3 min | 60℃ | 400℃ | 372 nm | 3.34eV |
| 3 | 0.5 (1:2 ratio) | 2hr | 0.3 min | 60℃ | 400℃ | 374 nm | 3.33eV |
| 4 | 1 (2:2 ratio) | 2hr | 0.3 min | 60℃ | 400℃ | 377 nm | 3.31eV |

**Peak wavelength:** To correlate the increase in band gap as concentration increases, the maximum wavelength of the UV vis spectras were determined and plotted, see figure s4.


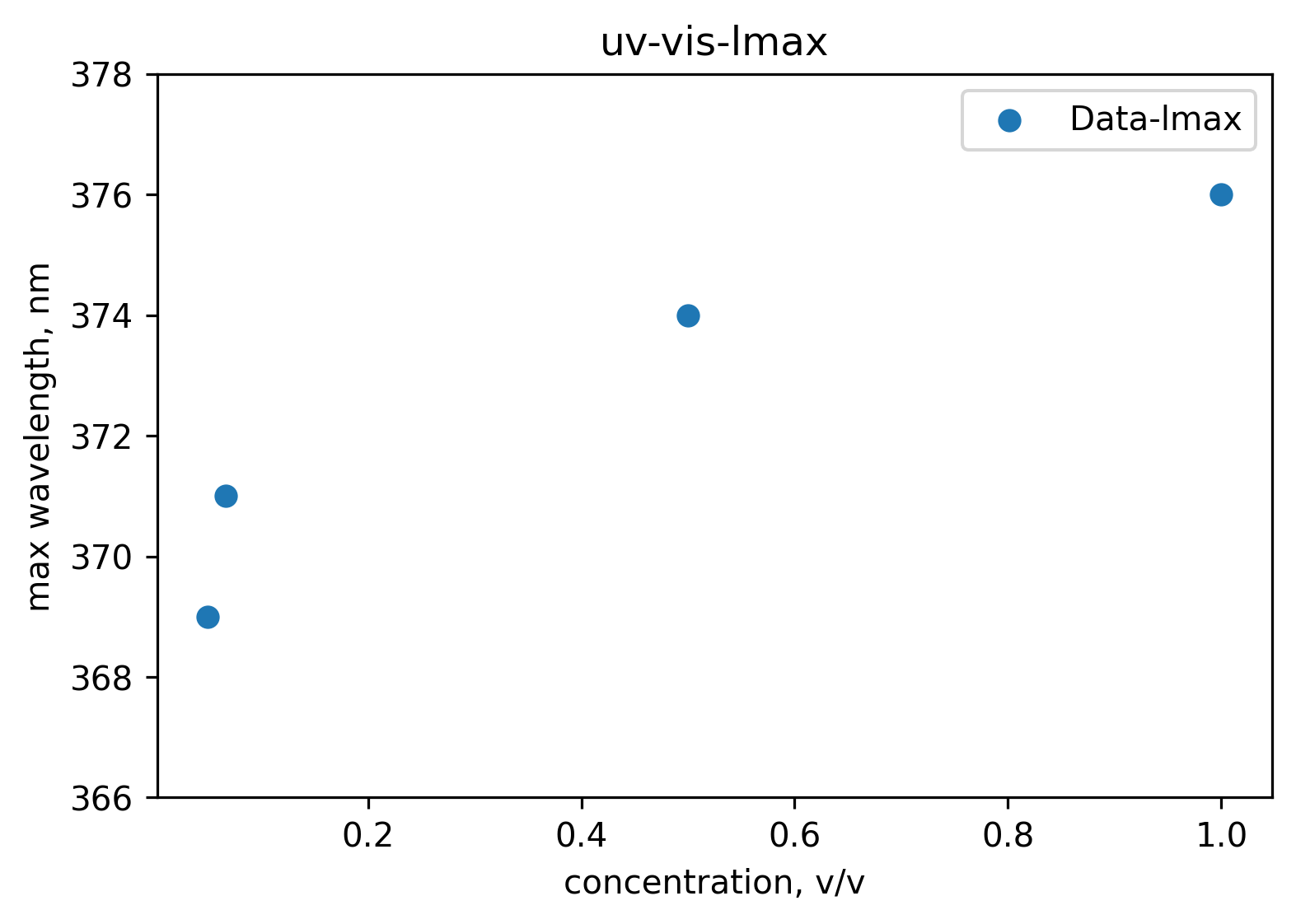


Figure s4. The peak wave length of the Uv vis spectra as a function of orange peel extract concentration

**FTIR spectra:** Table s3. Furrier transform infrared (FTIR) spectroscopy analysis

| **no** | **Functional group** | **Position** |
| --- | --- | --- |
| **Orange**  **peel** | (C-N), | 2924 cm-1 |
|  | C=O | 1739 cm-1 |
|  | C = C, C = O, stretching | 1640 cm-1 |
|  | N-H | 1427 cm-1 |
|  | C-H | 1059 cm-1 |
| **Zinc oxide NPs** | O-H stretching,phenols, alchols and water | 3414–3442 cm-1 |
|  | C=O stretching | 1400–1649 cm-1 |
|  | bending vibration of COH | 1398 cm-1 |
|  | the C-O stretching of esters and carboxylic functional groups | 1000 to 1300 cm-1 |
|  | Zn-OH stretching vibrations | 875 cm-1 and 712 cm-1 |

**XRD measurement:**

Table s4: Average crystalize sizes calculated from equation 2 of the the ZnO NPs synthesized using 1: 2 ratio of orange peel extract and zinc nitrate dehydrate.


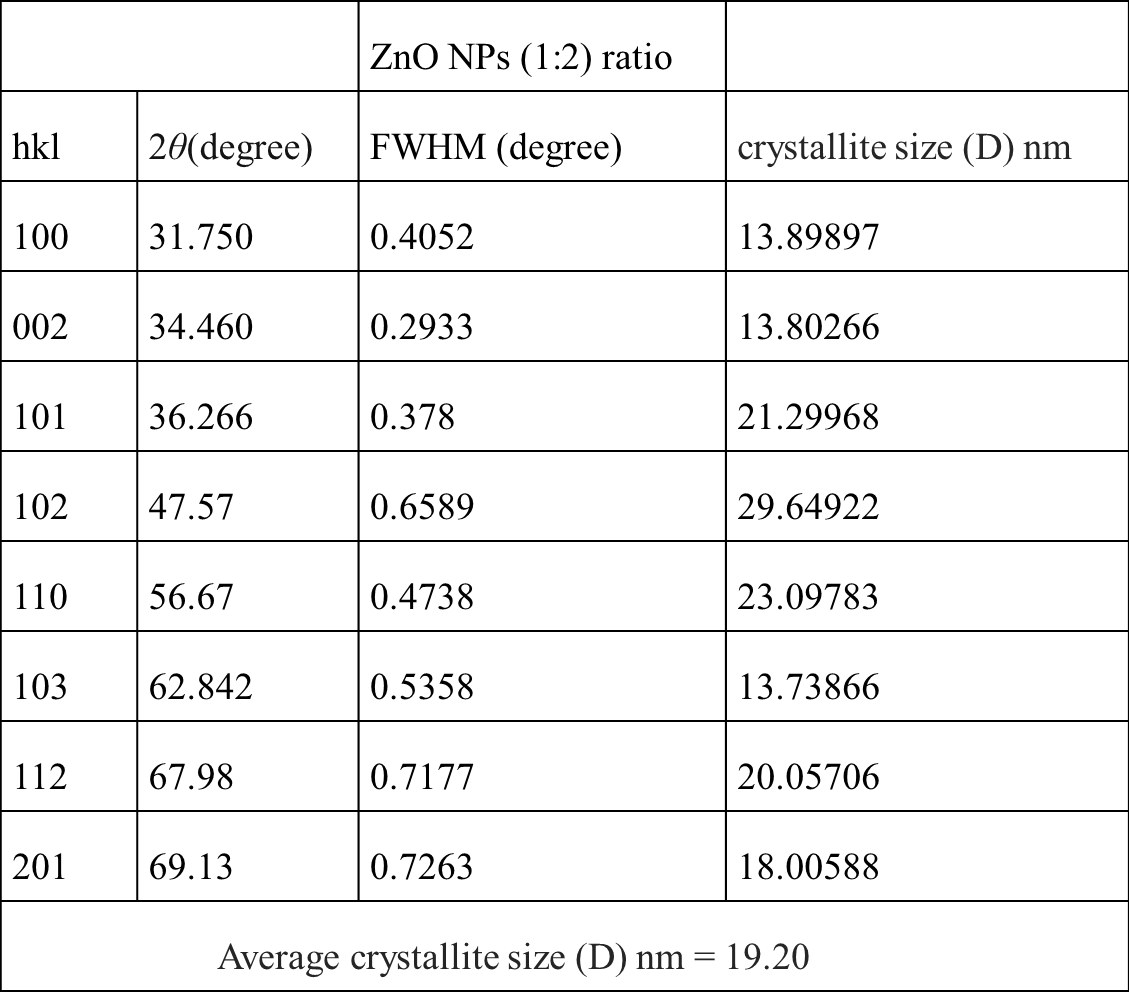


**Energy Dispersive X-ray Spectroscopy (EDX) Analysis:** Elemental analysis was performed using energy dispersive X-ray spectroscopy **(EDX),** and the results are summarized in Table s3.  The existence of zinc was confirmed by the presence of a few peaks between 1 and 10 Kev, including a significant peak at 1 keV, as shown in the EDX spectra (Figure s5 A). The zinc and oxygen elements are present with a weight percentage of 83.22% and 16.78%, respectively, which is close to the bulk weight percentage of zinc oxide (80 for Zn and 20 for O). In **addition, the analysis showed atomic percentages of 54.82% for zinc and 45.18% for oxygen,** with an atomic percentage composition similar to the results reported in related studies**. The distribution of elements within a sample is depicted in elemental mapping images, as shown in Figures s5 B and C.**The absence of any other elements suggests that the zinc oxide nanoparticles that were produced are extremely pure.


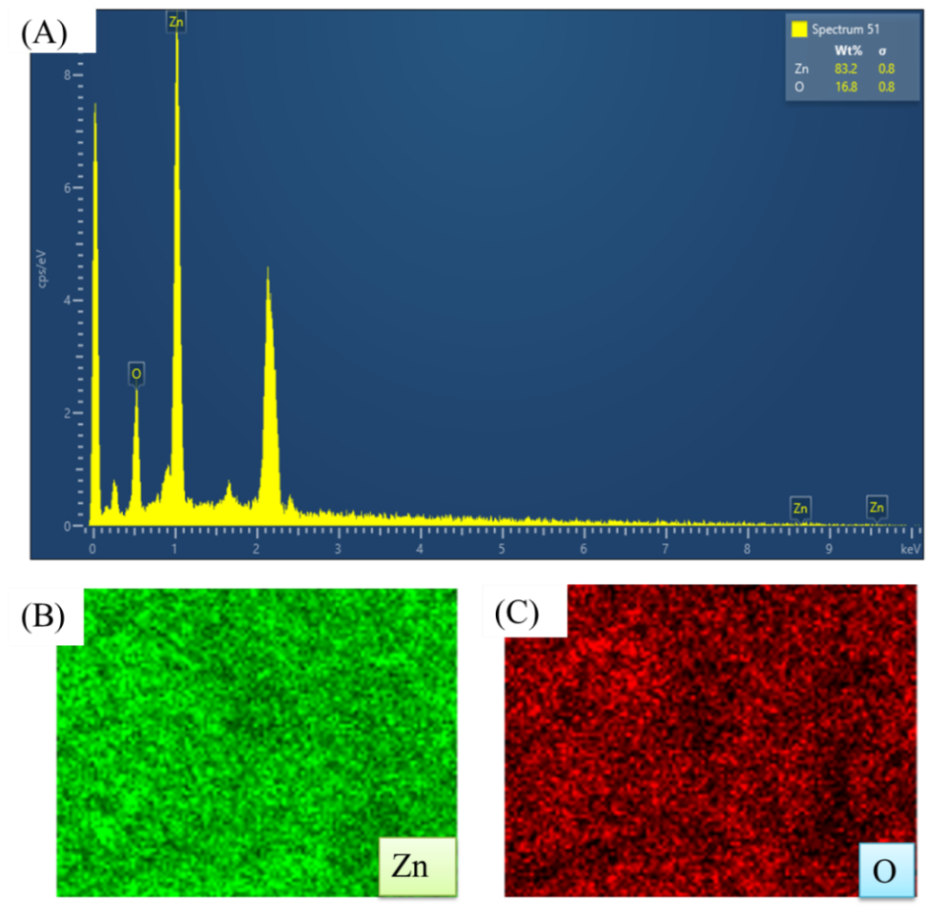


Figure s5: (A) EDX spectrum of ZnO NPs; (B and C) elemental mapping image the ZnO NPs.

Table s5: Elemental composition of ZnO NPs from the EDX analysis.


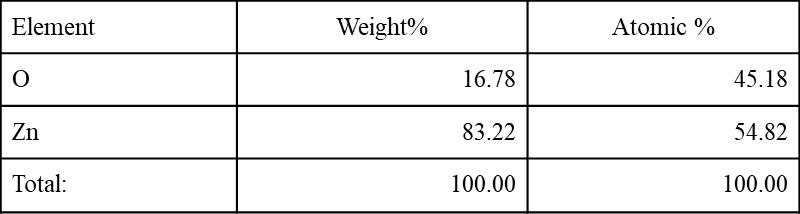


**References**

1. Srikant, V. & Clarke, D. R. On the optical band gap of zinc oxide. *J. Appl. Phys.* **83**, 5447–5451 (1998).

2. Hale, P. S. *et al.* Growth Kinetics and Modeling of ZnO Nanoparticles. **82**, (2007).

3. Ravindra, N. M., Ganapathy, P. & Choi, J. Energy gap-refractive index relations in semiconductors - An overview. *Infrared Phys. Technol.* **50**, 21–29 (2007).

4. The, I. Surfaces 2.1.

5. Shaba, E. Y., Jacob, J. O., Tijani, J. O. & Suleiman, M. A. T. *A Critical Review of Synthesis Parameters Affecting the Properties of Zinc Oxide Nanoparticle and Its Application in Wastewater Treatment*. *Applied Water Science* vol. 11 (Springer International Publishing, 2021).

6. Mamouei, M., Budidha, K., Baishya, N., Qassem, M. & Kyriacou, P. A. An empirical investigation of deviations from the Beer–Lambert law in optical estimation of lactate. *Sci. Rep.* **11**, 1–9 (2021).

7. Chem, J. M. Light absorption by colloidal semiconductor quantum dots. 10406–10415 (2012) doi:10.1039/c2jm30760j.
